# Treatment Outcomes of Tuberculosis Among Artisanal and Small-Scale Miners in Zimbabwe: A Follow-Up Observational Study Using Secondary Data

**DOI:** 10.3390/ijerph21101282

**Published:** 2024-09-26

**Authors:** Dingani Moyo, Fungai Kavenga, Ronald Thulani Ncube, Florence Moyo, Nathan Chiboyiwa, Andrew Nyambo, Godknows Madziva, Mpokiseng Ncube, Orippa Muzvidziwa, Tafadzwa Mperi, Blessings Chigaraza, Victoria Varaidzo Chizana, Plassey Ropafadzo Chinove, Frank Mudzingwa, Kudzaishe Mutungamiri, Collins Timire

**Affiliations:** 1 Baines Occupational Health Services, Harare 024, Zimbabwe; florencem@bainesohs.org (F.M.); godknowsm@bainesohs.org (G.M.); mpokisengn@bainesohs.org (M.N.); orippam@bainesohs.org (O.M.); tafadzwam@bainesohs.org (T.M.); blessingsc@bainesohs.org (B.C.); 2 Faculty of Medicine, National University of Science and Technology, Bulawayo 029, Zimbabwe; 3 Occupational Health Division, School of Public Health, University of the Witwatersrand, Johannesburg 2000, South Africa; 4 College of Health Sciences, University of Kwa-Zulu Natal, Durban 4001, South Africa; 5 Ministry of Health and Child Care, Harare 024, Zimbabwe; drkav8@gmail.com (F.K.); nathychiboyiwa@gmail.com (N.C.); andrewnyambo@gmail.com (A.N.); prchinhove@gmail.com (P.R.C.); collinstimire2005@yahoo.com (C.T.); 6 The Union Zimbabwe Trust, Harare 024, Zimbabwe; rncube@uzt.org.zw (R.T.N.); vchizana@uzt.org.zw (V.V.C.); 7 Faculty of Health Sciences, Zimbabwe Open University, Harare 024, Zimbabwe; 8 Hospice and Palliative Care Association of Zimbabwe, Harare 024, Zimbabwe; frank@hospaz.co.zw; 9 Jointed Hands Welfare Organization, Gweru 054, Zimbabwe; kmutungamiri@jointedhands.org

**Keywords:** TB treatment outcomes, artisanal and small-scale miners, gold mining, key population and vulnerable population

## Abstract

In Zimbabwe, artisanal and small-scale miners (ASMs) are a key vulnerable group with high risk for tuberculosis (TB), HIV, and silicosis. The main purpose of this study was to investigate treatment outcomes of TB among ASMs. We conducted a follow-up observational study using secondary data. We analyzed data from 208 ASMs treated for TB at two occupational health clinics. We found a high treatment success rate of 87%, comparable to the national average for drug-sensitive TB. Unsuccessful outcomes were due to death (5%) and loss to follow-up (7%). Over a quarter of ASMs had unknown HIV status. Our study is the first to document treatment outcomes of TB among ASMs in Zimbabwe. Encouragingly, this study demonstrates the possibility of achieving good TB treatment outcomes even among highly mobile populations like ASMs. Further research is needed to analyze leakages across the whole TB patient pathway among ASMs. Additionally, addressing the high rate of unknown HIV statuses among ASMs is crucial to further improve overall TB treatment outcomes in this population.

## 1. Introduction

Tuberculosis (TB) is a preventable and usually curable disease caused by a bacterium called Mycobacterium tuberculosis [1]. Worldwide, 10.6 million people are estimated to have fallen ill with TB in 2022, with 1.13 million associated deaths and with males disproportionately affected [1,2]. Sub-Saharan Africa (SSA) contributes 25% of the global TB burden [3]. Globally, the treatment success rate for drug-sensitive TB is 88% [1]. Furthermore, in Africa, the TB success rates are similar to the global average [1]. TB prevalence in miners in the region is estimated to be around 3000–7000 per 100,000 population, which is about 3 to 10 times higher than in the general population [3,4]. Artisanal and small-scale miners (ASMs) are vulnerable to occupational lung diseases (OLDs) such as TB and silicosis [3]. This is attributed to exposure to silica dust, overcrowding, poor ventilation in mines, HIV infection, alcohol abuse, poor nutrition, use of crude mining methods, and poor access to healthcare [3,5]. The high prevalence of TB in mining led to the signing of the Southern Africa Development Community (SADC) Declaration on TB in the mining sector (2012), the Framework for Harmonized Management of TB (2014), and the SADC Code of Conduct on TB in the mining sector (2015) to support a regional TB response in the mining sector [4].

Zimbabwe has over half a million ASMs who are actively involved in mining and also provide economic support to an estimated 2 million people [5]. According to the Global TB Report 2023, Zimbabwe has an estimated TB incidence of 33,000, with a 90% success rate for drug-sensitive TB and 42% for multi-drug-resistant TB (MDR-TB) [1]. Apart from studies conducted by Moyo et al., there is a paucity of studies describing the burden of TB, HIV, and silicosis amongst ASMs in Zimbabwe [5,6,7] The prevalence of TB in Zimbabwe among ASMs is 7400 per 100,000 which is more than 30 times greater than in the general population [6]. Studies conducted in Zimbabwe have revealed a triple burden of TB, HIV, and silicosis at 7%, 18%, and 19–21%, respectively, among ASMs [5,7]. Another study highlighted that 52% of ASMs had limited knowledge of HIV, an important risk factor for TB [8]. Silicosis and HIV comorbidity increase the risk of developing TB by fifteen-fold [9]. Silicosis is a chronic, progressive, and irreversible fibrotic lung disease. Studies have found that silicosis is highly prevalent in Zimbabwe, affecting 18–21% of ASMs [5,6]. The literature has revealed that silica dust exposure and/or silicosis increases the risk of TB by three- to four-fold [9,10,11]. The presence of silicosis can negatively affect the response of pulmonary TB to the prescribed chemotherapy, leading to frequent drug reactions and a higher risk of relapse compared to non-silicosis patients [12]. HIV, silicosis, and risk determinants for TB such as alcohol use and smoking are prevalent among ASMs and are known risk factors for unsuccessful TB outcomes [12,13,14,15,16,17]. Effective TB control is possible through early diagnosis and starting ASMs on effective TB treatment, as espoused in the first pillar of the End TB strategy. However, delays in receiving a diagnosis and starting TB treatment have to be taken into consideration among ASMs. This is due to several factors, ranging from long distances to health facilities, lack of consultation funds and medicine stock-outs [8]. For those who start TB treatment, adherence is challenging due to the hypermobile nature of ASMs, while silico-TB and HIV infection are known risk factors for poor TB treatment outcomes [12]. To date, there is scarce evidence on treatment outcomes among ASMs. 

To address OLDs including silicosis and TB among ASMs, there was a collaboration between the Ministry of Health and Child Care and Baines Occupational Services through the Kunda-Nqob’i TB (KNTB) Project supported by the United States Agency for International Development (USAID) in eight high-TB-burden priority districts in Zimbabwe [6]. The project established two occupational health clinics (OHCs), one at Gweru Provincial Hospital in the Midlands province and the other at Gwanda Provincial Hospital in Matabeleland South province [5,7]. Screening at artisanal and small-scale mining sites (workplace-based screenings), the project supported the deployment of radiological services to primary health services (expanded access to radiological services) to screen ASMs for OLDs including silicosis, TB, and HIV [6].

Given the high prevalence of TB in ASMs and their significant hypermobility and operation in remote and hard-to-reach areas, this study aims to evaluate treatment outcomes of TB among ASMs.

## 2. Materials and Methods

### 2.1. Study Design

A follow-up observational study using secondary data was conducted among ASMs. We reviewed medical records and TB registers for all ASMs who attended the OHCs in Gwanda and Gweru Provincial Hospitals under the USAID-supported KNTB project from 1 October 2022 to 30 September 2023. TB diagnoses were made at the OHCs, and treatment was initiated at the TB clinics within the provincial hospitals. 

### 2.2. General Setting

Tuberculosis remains a disease of public health concern in Zimbabwe, with the country having a dual burden of TB/HIV and drug-resistant (DR) TB. The Zimbabwe population-based impact assessment 2020 reported an HIV prevalence of 12.9% in adults aged 15 years and older, while Matabeleland South and Midlands Provinces had a HIV prevalence of 17.6% and 13.4%, respectively [18]. According to the Global TB Report 2023, Zimbabwe has a TB incidence rate of 204 per 100,000 population [1]. In Zimbabwe, TB treatment outcomes for new and relapse TB patients stand at 90% treatment success [1]. 

### 2.3. Specific Setting

We conducted this study at Gwanda and Gweru Provincial Hospitals in Matabeleland South and Midlands Provinces in Zimbabwe. The seven districts in these provinces which are implementing the USAID-supported KNTB project include the Gweru, Shurugwi, Kwekwe, Chirumanzu, Zvishavane, Insiza, and Gwanda districts, with two occupational health clinics at Gwanda and Gweru Provincial Hospitals. These districts have high artisanal and small-scale mining activities. Approximately 1560 ASMs were screened for TB at the two OHCs in the period between 1 October 2022 and 30 September 2023. 

### 2.4. Tuberculosis Screening and Diagnosis Method 

The OHCs are dedicated facilities that cater for ASMs, offering free services that include TB, HIV, silicosis screening, diagnosis, and linkage to care supported by the KN-TB project. All ASMs attending the OHCs were screened for TB and silicosis using chest radiographs and a TB symptom-screening tool. Diagnosis of TB was based on a positive result on Xpert mycobacterium tuberculosis rifampicin resistance (MTB/RIF) Ultra (Cepheid, Sunnyvale, CA, USA)/Truenat MTB/MTB Plus assays or clinical findings as per the Zimbabwe Tuberculosis and Leprosy Management Guidelines [19,20]. The diagnosis of silicosis was based on a threshold profusion of ≥1/0 on the International Labour Organization (ILO) International Classification of Radiographs for Pneumoconises. The chest radiographs were read by medical officers trained in the diagnosis of OLD and TB and were further checked by specialist occupational physicians experienced in the ILO classification of chest radiographs. Chest radiographs were performed using the Appelum digital X-ray machine 2010 u [21]. The diagnosis of HIV status was based on the OraQuick self-test (Orasure Technologies Inc., Bethlehem, PA, USA), Determine, Chembio, and Insti test kits, documented previous test results as well as self-reported HIV status. Reactive Oraquick tests were referred to an opportunistic infection clinic for confirmatory HIV testing according to the national HIV testing algorithm [22]. 

### 2.5. Definition of Treatment Outcome Variable

Patient treatment outcomes were based on the current Zimbabwe National Tuberculosis and Leprosy Management Guidelines, 6th Edition, May 2023 [19] (Table 1).

### 2.6. TB Screening and Diagnosis Process Flow for ASMs at Gweru and Gwanda Occupational Health Clinics

ASMs accessed the OHC through self-referrals, referrals by community-based volunteers, peer-to-peer referrals, and referrals by other health facilities. During consultations at the OHCs, ASMs underwent routine observations, history taking, and chest radiographs. Where indicated, ASMs underwent spirometry and audiometry testing. All ASMs presumed of TB had their sputum samples collected for bacteriological confirmation using the Xpert MTB/RIF Ultra assay. Voluntary HIV testing and counseling services were offered to all ASMs accessing services at the OHCs. ASMs with other medical conditions were linked to care within the hospital. 

### 2.7. Study Population

The study population comprised all records of all ASMs diagnosed with TB accessing services at the two OHCs. 

### 2.8. Sampling Procedure and Sample Size 

**Figure 1:**
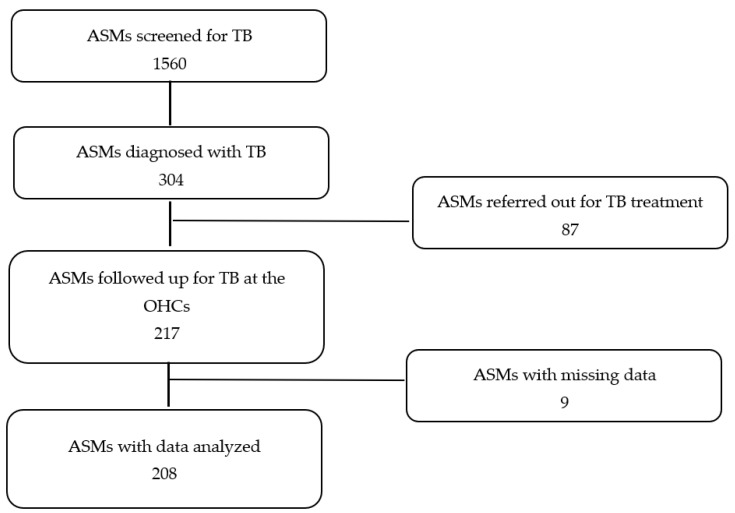
Sampling procedure for study participants for the two OHCs.

The sampling procedure for study participants was as shown in Figure 1 below. We collected data from the two OHCs. In these facilities, 304 ASMs were diagnosed with TB. Of these, we excluded 87 records for ASMs who were referred for TB treatment and care outside the facilities. A further nine records were excluded for missing data in most of the variables. A total of 208 records were included in this study for analysis. During this period, no DR-TB cases were diagnosed; hence, all recruited patients had drug-susceptible TB.

### 2.9. Data Collection 

Study participants’ data on socio-demographics (sex, age, and experience as an ASM), pre-existing medical conditions, previous history of TB diagnosis, and current data on TB diagnosis and treatment were extracted from paper-based individual client forms, which were available at the OHCs. Treatment outcomes and HIV status were extracted from the TB register. Data triangulation for all variables was performed using the Ministry of Health and Child Care (MoHCC) TB registers and the occupational health attendance registers. 

### 2.10. Permission and Ethical Approval 

Permission to conduct this study was obtained from the Permanent Secretary of the MoHCC and the national leadership of the artisanal and small-scale mining association. Ethical approval was obtained from the Medical Research Council of Zimbabwe (MRCZ) (Approval number: MRCZ/E/365). Confidentiality was ensured by excluding personal identification details from the final database. 

### 2.11. Data Analysis

Individual-level data were cleaned in MS Excel version 2013 and imported in Stata v 13.0 (StataCorp, College Station, TX, USA) [14] for further cleaning and analysis. Categorical variables, e.g., sex and history of TB, were summarized using frequencies and proportions. Continuous variables, such as age and experience as a miner, were assessed for normality by plotting histograms and summarized using means and standard deviations. TB treatment outcomes were categorized into unsuccessful (death, loss to follow-up, treatment failure, and not evaluated) and successful outcomes (cured and completed treatment). The key outcome variable was unsuccessful outcome. The association between unsuccessful outcomes with sociodemographic and clinical factors was assessed using Log binomial regression. Results were presented as relative risks and adjusted relative risks at 95% confidence intervals.

## 3. Results

We included 208 ASMs in the final database. There was a predominance of men, with 203 individuals (98%). The 35–44-year-old age group contributed the highest proportion of TB cases among ASMs, at 90 (43%) as shown in Table 2. Over 25% (56 out of 208) had an unknown HIV status. There was a high proportion of risk determinants for TB such as smoking (57%) and alcohol use (57%). The majority of ASMs had symptomatic TB, with 194 (93%) presenting with a cough. Bacteriologically confirmed TB cases among ASMs were low, at 51%. Over half of the ASMs had silicosis. 

Overall, successful treatment outcomes were recorded in 180 (87%) [95% CI: (82–91%)] ASMs. Death and LTFU accounted for 93% (26/28) of unsuccessful outcomes as shown in Table 3. 

The risk of unsuccessful outcomes was high among ASMs who had an unknown HIV status, alcohol use, smoked, and had low mining experience of <5 years as shown in Table 4. However, none of the factors were significantly associated with unsuccessful outcomes. 

## 4. Discussion

To our understanding, this is the first study to document treatment outcomes of TB among key vulnerable population groups such as ASMs in Zimbabwe. We observed a high TB treatment success rate of 87%. Although the risk for unsuccessful outcomes was high among people who reported a mining experience of <5 years and those with unknown HIV status, none of these factors were significantly associated with unsuccessful outcomes. 

The high treatment success reported in this study is a commendable finding and is comparable to the treatment success reported globally. Globally, treatment success among people with drug sensitive (DS) TB is around 86% and 90% in Zimbabwe [1]. Considering that ASMs are a highly mobile group, these results show that it is possible to attain comparable outcomes in this vulnerable population. A previous study by Moyo et al., 2023, showed that ASMs were willing to seek medical services when they were accessible [8]. The comparable rate of TB treatment outcomes with that of the general population is likely due to the cessation of mining activities by the affected ASMs who return to their usual places of residence and access healthcare facilities just like the general population. Furthermore, the USAID KNTB project has conducted several awareness campaigns on TB screening and treatment, as well as supporting free access to TB screening, and health and safety education through the mobile health outreach facilities in the project districts. The establishment of OHCs under the KNTB project has improved access and availability of TB, HIV, and silicosis screening and treatment services among ASMs [6]. 

The relatively higher risk of unsuccessful outcomes among ASMs with mining experience of less than 5 years warrants further exploration. It is likely that ASMs with less than five years of experience could have been of a lower socioeconomic status when compared to miners with more experience, whose economic status would be better. In line with this, several studies have revealed poor TB treatment outcomes that were positively associated with lower socioeconomic status [23,24,25]. Furthermore, in the first few years of entry into artisanal mining, ASMs are required to secure independent mining sites, and this may involve spending prolonged periods of time in remote areas that are underserved in terms of health services, resulting in challenges in accessing health services. Travel costs to healthcare facilities are generally a challenge for ASMs and worse so for early-career ASMs who are yet to establish themselves, and this could account for the observed unsuccessful TB treatment outcomes. A lack of experience, an increased exposure to silicon dioxide, and an inadequate implementation of the hierarchy of health and safety controls could account for the observed poor treatment outcomes in these ASMs.

In this study, a high proportion of ASMs had an undocumented HIV status. The study by Moyo et al. (2022) revealed a high proportion of unknown HIV status among ASMs [5]. Voluntary HIV counseling and testing is routinely offered at the OHCs, and patients are required to give consent. TB/HIV co-infection is a risk factor for unsuccessful TB treatment outcomes [26,27]. Hence, a high proportion of ASMs with an unknown HIV status is worrisome. This might be due to ASMs not consenting to HIV testing or to incomplete medical records with undocumented HIV status. There is a need to strengthen recording and reporting systems at the facility level. It has been found that good recording and reporting systems at the health facility level are key to ensuring the completeness of data [28]. HIV testing is the rate-determining step for linkage to HIV prevention, treatment, and care, including access to TB preventive therapy (TPT) for people living with HIV (PLHIV) without signs and symptoms of TB. This reduces the transmission of HIV, incidence of TB, and morbidity and ultimately improves TB treatment outcomes

Similar to the results of our study, previous studies in ASMs have shown a gender disproportion, with male dominance in the mining industry [5,6,7,16]. This is in line with TB notification trends in Zimbabwe, where males contribute about two-thirds of the disease burden [1]. Contrary to a previous study that highlighted the association between smoking, alcohol use, and poor TB treatment outcomes [14], in our study, this association was not statistically significant. Our study did not comprehensively assess the effects of alcohol and smoking use during TB treatment on treatment outcomes as historical data were used. 

The majority of the study participants had over 10 years of mining experience, showing that a longer duration of silica dust exposure due to limited use of proper personal protective equipment increases the risk of TB among ASMs. Rupani et al. observed that people with silico-tuberculosis have a 2.3 times higher risk of developing unsuccessful TB treatment outcomes [12]. Our study, however, did not elicit that association but rather the opposite. This is most likely due to the implementation of the USAID-supported KNTB project, which has supported extensive awareness campaigns on TB prevention and treatment, silicosis prevention, the relationship between silica dust exposure and TB, and healthy and safe mining practices in the artisanal and small-scale mining project districts. Death is a hard outcome. In subgroup analysis, Rupani et al. observed that people with silico-TB had 3 times higher odds of death due to TB than those diagnosed with TB alone [12]. Our subgroup analysis (using death vs survival as outcome) did not yield statistical significance, which is attributed to low numbers. This warrants a further study with a larger cohort of ASMs. Although more than half of the TB cases were bacteriologically confirmed, the proportion fell short of the WHO-recommended target of 60% [19]. 

The high burden of TB among ASMs is driven by several factors, including excessive exposure to silicon dioxide during mining, poor TB knowledge, attitudes, and practices, and associated risk factors such as HIV, silica dust exposure, silicosis, and smoking, among many other factors. The implementation of healthy and safe mining practices, such as control of silica dust exposure, and increased awareness of the relationship between TB, silicosis, and HIV among ASMs are key in reducing the burden of TB in this vulnerable population. 

This study had limitations. Firstly, the study population was recruited from OHCs, which is not representative of all ASMs. In addition, outside the study districts, there are no available TB data that are routinely collected and specific to ASMs. Secondly, this study relied on secondary data, which might have missed important variables affecting data quality and completeness, limiting the scope of the analysis. Thirdly, there is potential for survival bias. ASMs who started treatment may have better health-seeking behavior than those who delay seeking treatment and may die before they commence treatment. Sicker ASMs are more likely to relocate to their relatives in search of family support and carers. We also lack information about the cascade of care from screening to TB diagnosis, including the duration from the onset of symptoms to starting TB treatment. Furthermore, the other limitation was that our study lacked a comparable group. Future research should investigate upstream activities such as the TB cascade from TB screening, diagnosis, and care, including TB and HIV collaboration among ASMs to identify leakages along the care cascade. Despite the limitations of this study, this is the first-ever study of its kind in Zimbabwe. This study was exploratory and had important findings demonstrating good treatment outcomes in a highly mobile population that has often been thought to have poor health-seeking behavior. Institutionalized OHCs are a novel approach to improving access to differentiated services and care for ASMs. Our study has provided very important insights into TB treatment outcomes among ASMs. 

## 5. Conclusions

This study adds to the relatively new body of knowledge on TB treatment outcomes among ASMs. It shows that it is possible to attain high treatment success among ASMs. However, more needs to be done to attain the global targets of at least 90% treatment success. While the treatment success rate (87%) is encouraging, addressing risk factors like unknown HIV status could further improve outcomes. Further research is needed to explore interventions that can effectively reduce the TB burden in this vulnerable population.

## Figures and Tables

**Table 1:** TB treatment outcomes.

	TB Treatment Outcome	Description
1.	Cured	A patient who completed treatment with evidence of bacteriological response and no evidence of failure.
2.	Treatment completed	A patient who completed treatment but whose outcome does not meet the definition of cure or treatment failure.
3.	Treatment failed	A patient whose treatment regimen needed to be terminated or permanently changed to a new regimen or treatment strategy.
4.	Died	A patient who died for any reason before starting treatment or during treatment.
5.	Lost to follow-up	A patient who did not start treatment or whose treatment was interrupted for 2 consecutive months or more.
6.	Not evaluated	A patient for whom no treatment outcome was assigned. This includes cases transferred out to another treatment unit as well as cases for whom the treatment outcome is unknown to the reporting unit.
7.	Treatment success	This category is the sum of cured and treatment completed.

**Table 2:** Sociodemographic and clinical characteristics of artisanal miners who were enrolled in this study.

Characteristic		N (%) ⁑
Total		208
Sex	Male	203 (98)
	Female	5 (2)
Age category	18–24	8 (4)
	25–34	71 (34)
	35–44	90 (43)
	45–54	26 (13)
	55+	13 (6)
HIV status	Positive	73 (35)
	Negative	79 (38)
	Unknown	56 (27)
Risk determinants for TB		
Smoking status	Yes	118 (57)
Alcohol use	Yes	118 (57)
Other chronic conditions ^†^	Yes	28 (13)
Previous history of TB	Yes	47 (23)
	No	159 (76)
	Not recorded	2 (1)
Type of pulmonary TB	Bacteriologically confirmed	107 (51)
	Clinically diagnosed	101 (49)
Diagnosed with silicosis	Yes	121 (58)
	No	87 (42)
Presenting signs, symptoms		
Cough	Yes	194 (93)
Shortness of breath	Yes	153 (74)
Experience as a miner (years)	<5	37 (18)
	5–9.9	56 (27)
	10–35	110 (53)
	Not recorded	5 (2)

⁑ = column percentage, † = conditions included asthma, hypertension, kidney disease, pneumonia, and diabetes mellitus.

**Table 3:** Treatment outcomes among artisanal and small-scale miners who were enrolled in this study.

Treatment Outcomes		N	(%) ^§^
Successful outcomes		180	(87)
	Cured	39	(19)
	Treatment completed	141	(68)
Unsuccessful outcomes		28	(13)
	Not evaluated/LTFU	16	(7)
	Died	10	(5)
	Failed treatment	2	(1)

§ = column percentage; LTFU = loss to follow-up.

**Table 4:** Factors associated with unsuccessful outcomes among ASMs who started TB treatment.

Factors		Total	Unsuccessful Outcomes	
			n (%) ^‡^	RR (95% CI)
		208	28 (13)	
Age category	18–24	8	2 (25)	2.25 (0.59–8.55)
	25–34	71	9 (13)	1.14 (0.49–2.66)
	35–44	90	10 (11)	Ref
	45+	39	7 (18)	1.62 (0.66–3.93)
HIV status	Positive	73	7 (10)	0.69 (0.28–1.68)
	Negative	79	11 (14)	Ref
	Unknown	56	10 (18)	1.28 (0.58–2.81)
Previous TB (n = 206)	Yes	47	6 (13)	0.97 (0.41–2.27)
	No	159	21 (13)	Ref
Smoking status (n = 206)	Yes	118	14 (12)	0.76 (0.38–1.52)
	No	90	14 (16)	Ref
Alcohol use (n = 206)	Yes	118	14 (12)	0.75 (0.38–1.48)
	No	88	14 (16)	Ref
Other chronic conditions *	Yes	28	3 (11)	0.77 (0.25–2.39)
	No	180	25 (14)	Ref
Mining experience (years) (n = 203)	10–35	110	13 (12)	0.62 (0.27–1.50)
	5–9.9	56	8 (14)	0.76 (0.30–1.91)
	<5	37	7 (19)	Ref
Diagnosed with silicosis	Yes	121	14 (12)	0.72 (0.36–1.43)
	No	57	14 (16)	Ref

‡ = row percentages; RR = relative risk; CI = confidence interval; TB = tuberculosis; * conditions included asthma, hypertension, kidney disease, pneumonia, and diabetes mellitus.

## Data Availability

The data presented in this study are available on request from the corresponding author. The data are not publicly available due to authorizations that may be required by the Ministry of Health and Child Care of Zimbabwe.
